# Acute and Chronic Catabolic Responses to **CrossFit^®^ and Resistance Training in Young Males**

**DOI:** 10.3390/ijerph17197172

**Published:** 2020-09-30

**Authors:** Emanuela Faelli, Ambra Bisio, Roberto Codella, Vittoria Ferrando, Luisa Perasso, Marco Panascì, Daniele Saverino, Piero Ruggeri

**Affiliations:** 1 Department of Experimental Medicine, Università degli Studi di Genova, 16132 Genoa, Italy; emanuela.faelli@unige.it (E.F.); ambra.bisio@unige.it (A.B.); vittoriaferrando1@gmail.com (V.F.); luisa.perasso@unige.it (L.P.); daniele.saverino@unige.it (D.S.); ruggeri@unige.it (P.R.); 2 Centro Polifunzionale di Scienze Motorie, Università degli Studi di Genova, 16132 Genoa, Italy; marco.panasci87@gmail.com; 3 Department of Biomedical Sciences for Health, Università Degli Studi di Milano, 20133 Milano, Italy; 4 Department of Endocrinology, Nutrition and Metabolic Diseases, IRCCS MultiMedica, 20138 Milano, Italy; 5 Department of Neuroscience, Rehabilitation, Ophthalmology, Genetics and Maternal Child Health, Università degli Studi di Genova, 16132 Genoa, Italy

**Keywords:** CrossFit, cortisol, interleukin 1-beta, uric acid, catabolic responses

## Abstract

Given the wide variety of conditioning program trainings employed, the present study compared the catabolic effects induced by CrossFit^®^ and resistance training in moderately trained subjects. Twenty males joined either the CrossFit^®^ group (n = 10; 30 min/day of “workout of the day”) or the resistance training (RT) group (n = 10; 30 min/day of resistance exercises) thrice a week, for 8 weeks. Salivary levels of cortisol, interleukin 1-beta (IL-1β), and uric acid were assessed via enzyme-linked immunosorbent assays before (PRE) and 30-min after (POST) SESSION 1 and SESSION 24. Variables’ percentual changes were computed as (POST-PRE)/PRE*100 in each session (Δ%). CrossFit^®^ acutely increased cortisol levels in both sessions, with a significant decrease in Δ%cortisol from SESSION 1 to 24. In the RT group, cortisol values decreased in both sessions, only acutely. A significant decrease in IL-1β levels was registered acutely in both groups, in both sessions, whereas Δ%IL-1β was not different between the two groups. While uric acid levels increased in both groups acutely, a chronic downregulation of Δ%uric acid, from SESSION 1 to 24, was appreciated for the RT group only. Overall, CrossFit^®^ appeared to induce more intense effects than the RT program as to the investigated catabolic responses.

## 1. Introduction

Successful sports performance entails an optimal combination of aerobic and anaerobic metabolism, muscular power and strength, speed and agility, according to specific tasks [1]. Amongst a variety of training regimens for increasing performance, resistance training (RT) is essential, as it enhances muscular strength and power [2]. Typically, RT aims at increasing skeletal muscle strength by working against a weight or force. Recently, high-intensity functional training (HIFT) has received growing popularity and is alleged to improve overall physical conditions [3]. HIFT relies on basic elements of “every day” movements derived from both aerobic and resistance efforts, performed at high intensities. The efficiency of exercise training depends not only to the training load, but also on the athlete’s capability to sustain it. One way to gauge exercise-induced internal environmental stress fluctuations is through the evaluation of the hormonal responses, and through the monitoring of biomarkers of inflammation and oxidative stress. Improving overall performance or accounting for residual training effects might rely on reproducible indicators of reactions to training. Along this line, any effort made to quantify the fine balance between training practice and athlete’s tolerance may help to optimize training programs [4]. 

CrossFit^®^ is one of the new modality of HIFT that have emerged in the last few years. A typical CrossFit^®^ training is organized into daily sessions called “workouts of the day” (WOD), including metabolic exercises (running, rowing), gymnastic movements (pull-up, push-ups, air squats, burpees), and weightlifting (snatch, clean, and jerk) and performed at an intensity close to 95% of the maximum heart rate (HRmax) [3,5]. WODs are organized as circuits with little or no rest periods, performed “as many repetitions as possible” (AMRAP) during a given time domain [3] or as quickly as possible over periods of 10 to 20 min [6].

The undoubted beneficial effects of exercise have been underlined in a number of studies. Nevertheless, exercise is a stress situation that challenges homeostasis [4], and the body must find a new dynamic equilibrium, that requires, among others, adaptive responses of the hormonal, metabolic, and immune systems. As concerns the outcomes of HIFT programs, CrossFit^®^ has been demonstrated to improve body composition and physical fitness [6,7], also eliciting metabolic [8], inflammatory [8,9,10], and hormonal responses [11]. Furthermore, CrossFit^®^ training has been shown to induce an immunosuppressive effect [12] and acute oxidative stress responses, affecting the immune system [13]. Particularly, it has been shown that a HIFT with short rest protocol carried out in men and women with no experience in resistance training elicits significant increases in inflammation and induced hyperreactions in metabolic and adrenal (cortisol) functions. Another study showed that two consecutive HIFT sessions increase pro/anti-inflammatory cytokines with no interference on muscle performance in the recovery period [8]. On the whole, a recent review [14] analyzed the prevalence and incidence of physiological responses and chronic adaptations to HIFT programs, which resulted in increased acute oxidative, metabolic, cardiovascular, and hormonal stress, depending on the protocol adopted, as for intensity, duration, and training status of the subjects. Interestingly, the authors reported that an insufficient rest between HIFT sessions resulted in unfavorable cytokine responses, with a decrease in anti-inflammatory and increase in proinflammatory cytokines. We can assume that advanced-level technique during maximal timed exercise repetitions, without suitable rest intervals between sets and shifts, as well as an inadequate recovery time between high-volume loads and training sessions (such as CrossFit^®^) may produce premature fatigue and additional oxidative stress level in athletes [15]. The immune and endocrine systems are closely intertwined in modulating an appropriate response to physiological and psychological stress factors [16]. Moreover, biochemical monitoring is useful in sports contexts to assess and manage workload and fatigue of athletes at all levels [17]. With regard to exercise, cortisol plays an important regulatory role in metabolic responses to stressor events through the activation of energy proteolysis and lipolysis [18]. Lastly, the regulation of protein turnover during recovery from physical exercise, involving also contractile myofibrils adaptation to training, is closely linked to appropriate glucocorticoid actions [19]. Studies have shown significant elevations in acute cortisol secretion as no change [20] or reductions [21]. Elevations of cortisol levels have been reported during normal strength and power training [22], while in CrossFit^®^ training a greater acute cortisol response [11] and a lower chronic response were obtained compared to strength and power training [23]. Assessing adrenal function activation is relevant as exacerbated cortisol concentrations may lead to reiterative stress over subsequent training sessions, contributing to a non-functional overreaching or even to overtraining.

Yet, it is well established that training can alter host defense, leading to changes in disease susceptibility and severity [24]. Both aerobic and RT have been explored to understand their inflammatory mediators and the parameters of the reaction to exercise [25]. For instance, as to exercise-induced changes in interleukin-1 (IL-1) circulating levels, long-distance runners showed chronically elevated plasma IL-1 without an acute increase 3 h after an eccentric exercise bout, whereas their untrained controls had lower baseline IL-1 levels along with acute spurs 3 h post-exercise [26]. In line with this, a two-fold increase in plasma IL-1 beta β (IL-1β) concentrations were found 30 min after 45-min cycling exercise at 70% of VO_2_max in non-athlete subjects [27]. In another work, plasma IL-1 levels were undetectable after exercise [28]. 

Finally, the antioxidant defense is activated by exercise, preferably via low molecular weight non-enzymatic antioxidants, i.e., uric acid [29]. It is known that plasma urate levels increase with exercise, possibly as a physiological coping mechanism to increased oxidative stress. Recently it has been shown that anaerobic trainings [30], as well as a CrossFit^®^ program [13], induce oxidative stress immediately after the exercise, and also during the early period of recovery. However, the mechanisms controlling training load are not fully known, and stress responses are key determinants to that purpose. Given the complexity of these HIFT programs and the increasingly high number of its participants, studies are required to investigate the effects of these trainings and whether a tailored training optimization could be obtained.

The present study aimed at shedding light on the stress responses elicited by two strength/power programs in order to bring useful indications in the monitoring and scheduling of these two different training regimens, including their respective periodization plans. Therefore, the purpose of this study was to examine the oxidative stress, the hormonal and the immunological acute and chronic responses following 8 weeks of a CrossFit^®^ program and of a classical gym RT program in moderately trained subjects. Based on previous studies, the primary hypothesis was that these conditioning training programs would be accompanied by exacerbated stress responses. As a secondary endpoint, magnifying the extent of these different training-induced changes would be likewise of interest.

## 2. Materials and Methods

### 2.1. Participants

Twenty male young adults, classified as moderately trained according to Sheppard et al. [31], with 1 year of experience in CrossFit^®^ and Resistance Training, were enrolled for the study. They were divided into two groups, with a convenience sampling: the CrossFit^®^ group (n = 10) and the RT group (n = 10). Before and after the intervention period, participants underwent to the maximum repetition test (1RM test), in order to assess their individual maximum load lifted (see 1RM test paragraph for a description of the tests). The two groups were homogeneous regarding age (CrossFit^®^ group 24.6 ± 3.4 years, RT group 26.3 ± 3.6, t(18) = −1.09, *p* = 0.29), and practice experience (1 year). 

All subjects were fully informed about the study aims and procedures and gave their informed consent for inclusion before they participated in the study. The study was conducted in accordance with the Declaration of Helsinki, and the protocol was approved by the Ethics Committee of Università degli Studi di Milano (protocol number 52/20, attachment #4 of 14/05/20). 

All participants were instructed not to change their diet and physical activity practices throughout the intervention period. Subjects were also recommended not to consume alcohol, caffeine, theine, hot drinks, or smoke within 24 h of the session. None of the participants were using medications or performance-enhancing drugs during the study.

Exclusion criteria were muscle or joint injuries, orthopedic problems, severe visual impairment, or any other contraindication within three months before the commencement of the study.

### 2.2. Sample Size

Estimation of sample size was performed using the GPower software (3.1 software, Düsseldorf, Germany) applying ANOVA repeated measures (F Test). This calculation generated a desired sample size of at least 18 subjects. However, we recruited 20 participants, 10 in the CrossFit^®^ group and 10 in the RT group, to allow for drop-out during the intervention period [32]. 

### 2.3. Experimental Protocol

#### 2.3.1. Experimental Design

**Figure 1:**
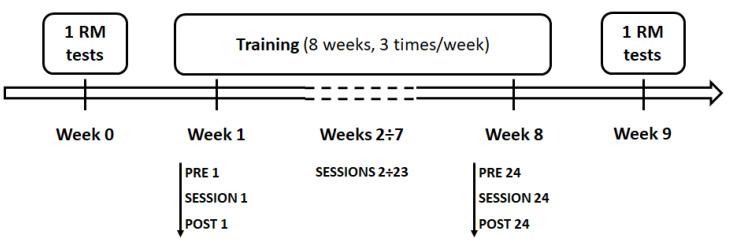
Experimental design. Before (Week 0, baseline measures) and after (Week 9, ending measures) the intervention period, participants underwent to the maximum repetition test (1RM test). Both CrossFit^®^ and resistance training (RT) lasted 8 weeks (3 times/week). Before (PRE) and 30 min after (POST) the end of the first and the last training sessions (SESSION 1 and SESSION 24, respectively), cortisol, IL-1 beta, and uric acid levels were measured.

Before and after the intervention period, participants underwent (a) the maximum repetition test (1RM test), in order to assess their individual maximum load lifted, and (b) body composition assessment. Intervention period lasted 8 weeks. Throughout the 8-week training program, participants performed 24 sessions, 3 times/week, and all the training sessions were held at the same time of day (5 pm). Before (PRE) and 30 min after (POST) the end of both the first training session (SESSION 1) and the last training session (SESSION 24), salivary levels of cortisol, IL-1 beta, and uric acid were ascertained (Figure 1). 

#### 2.3.2. RM Tests

Prior to 1RM tests, subjects performed 5 min of warm-up including low-intensity functional movements for the joints. The maximum repetition test was recorded as the maximum load lifted (1RM) for one repetition. 

Participants established one-repetition maximums (1RMs) on military press, squat clean, and power clean. Before attempting a 1RM, subjects performed a progressive series of five submaximal sets of 1 to 2 repetitions with moderate to heavy loads (50–90%) of the estimated 1RM. If a weight was properly lifted during a 1RM trial, the subsequent 1RM weight attempt was increased by 2.5 to 7 kg, and the participant attempted another 1RM trial with 3 min of rest between efforts. Each 1RM was determined within 3 to 5 trials [33,34]. 

#### 2.3.3. CrossFit^®^ Sessions

Each CrossFit^®^ session consisted of 20 min of warm-up (running at low intensity and joint mobility exercises), 30 min of WOD (metabolic + gymnastic + weightlifting), and 10 min of cool down. The WOD was characterized by metabolic (e.g., running, jumping rope), gymnastics (e.g., pull-ups, squats), and weightlifting (e.g., front squats, kettlebell swings) exercises [23]. 

Templates were individually prescribed and recorded for each CrossFit^®^ participant. 

The CrossFit^®^ protocol was as follows: 

Weeks 1–4: (metabolic): 4 min of running, 2 min of rest, 4 min of jumping rope; (gymnastic): 4 min of pull-ups and squats, 2 min of rest, 4 min of pull-ups and squats; (weightlifting): 4 min of front squats and kettlebell swings at 50–60% 1RM, 2 min of rest, 4 min of front squats and kettlebell swings at 50–60% 1RM. 

Weeks 5–8: (metabolic): 4 min of running, 2 min of rest, 4 min of jumping rope; (gymnastic): 4 min of pull-ups and squats, 2 min of rest, 4 min of pull-ups and squats; (weightlifting): 4 min of front squats and kettlebell swings at 65–75% 1RM, 2 min of rest, 4 min of front squats and kettlebell swings at 65–75% 1RM. 

The WODs were performed as quickly as possible with no rest period or “as many repetitions as possible” (AMRAP) fashion. All CrossFit^®^ sessions were held in a CrossFit^®^ training center and supervised by a CrossFit^®^ Level 1 certified trainer. Each training session lasted approximately 60 min, including warm-up and cool-down.

#### 2.3.4. Resistance Training Sessions

Resistance training sessions lasted approximately 60 min, with one minute of rest between all sets and exercises. Sessions always included 3 sets of 15 crunches and other exercises varied from Monday (i.e., bicep curls, lateral pulldowns) to Wednesday (i.e., triceps pulldowns, bench presses) to Friday (i.e., military presses, leg extensions, reverse leg curls, and seated leg presses). 

Participants performed three sets of each exercise, separated by 1-min rest, using the following progressive repetition scheme: Weeks 1–2: 15 reps at 50% 1RM; Weeks 3–4: 12 reps at 55% 1RM; Week 5: 10 reps at 60% 1RM; Week 6: 10 reps at 65% 1RM; Week 7: 8 reps at 70% 1RM; Week 8: 8 reps at 75% 1RM (Table 1).

### 2.4. Measures

#### 2.4.1. Saliva Sample Collection and Analysis

Saliva samples were collected before (PRE) and 30 min after (POST) the end of the first training session (SESSION 1) and the last training session (SESSION 24). Saliva samples were obtained by means of cotton swabs and saliva collecting tubes (SalivaBio Oral Swab, Salimetrics, Cambridge, UK). The athletes were instructed to place the cotton swab into their mouths, under the tongue, for 2 min. The absence of blood contamination was checked with a salivary blood contamination kit (Salimetrics LLC, Cambridge, UK). The saliva collecting tubes were centrifuged at 3000 rev/min for 15 min at 4 °C. Samplings were stored at −80 °C until they were assayed. To exclude inter-assay variance, all samples were thawed once and analyzed in triplicate in the same assay run. As said, subjects refrained from consuming any food, drinking hot fluids, or brushing their teeth for two hours prior to their arrival. Upon their arrival, before starting the training session, participants were asked to remain seated for 15 min before providing their resting sample (PRE), and subsequently, they completed the training session. At the end of the training session, they were asked to remain in a relaxed position (e.g., seating or standing) for 30 min until the collection time point (POST). The participants could drink water freely during resting time but were asked to abstain from drinking within 10 min of any post-exercise collection time point. Concentrations of cortisol, IL-1β, and uric acid were assessed via enzyme-linked immunosorbent assays (ELISA, Salimetrics LLC, Cambridge, UK, product code 1-3102, 1-3902, and 1-3802, respectively), following manufacturers’ instructions. For cortisol, the assay range was 0.012-3.000 µg/dL and the sensitivity <0.007 µg/dL, for IL-1β the range was 3.13–200 pg/mL and the sensitivity <0.37 pg/mL, and for uric acid the range was 0.07–5 mg/dL with a sensitivity of 0.07 mg/dL. All samples were tested in duplicate. Intra-assay deviation was <10% for all kits.

#### 2.4.2. Body Composition

Body composition was evaluated by using bioelectrical impedance analysis (BIA; Tanita, BC-420 MA). Weight (kg), body mass index (BMI, kg/m^2^), lean mass (kg), and fat mass (%) were considered.

### 2.5. Statistical Analyses

Dependent variables were checked for normal distribution by means of Shapiro-Wilk test and for sphericity violation by Mauchly’s test.

Repeated measure ANOVAs with EPOCH (2 levels, baseline and ending) as within-subjects factor, and GROUP (2 levels, CrossFit^®^ and RT), as between-subjects factor were applied to statistically evaluate changes in parameters defining the body composition (weight, body mass index (BMI), lean mass, and fat mass) and functional measures (1RM power clean, 1RM squat clean, and 1RM military press).

Cortisol and IL-1β values were normally distributed whilst uric acid was not. Uric acid distribution was normalized by applying a logarithmic transformation (log 10). Statistical analysis was performed in three phases. Variables’ changes were analyzed PRE and POST the first and last training sessions for both groups. Cortisol, IL-1β, and uric acid were analyzed by means of RM-ANOVA with SESSION (2 levels, SESSION 1 and SESSION 24) and TIME (2 levels, PRE and POST) as within-subjects factors.

Furthermore, to compare the amount of changes between PRE and POST values in the two SESSIONS between groups, we computed for each parameter the percentual changes as follows (POST-PRE)/PRE*100 in each session and group. The obtained Δ%cortisol, Δ%IL-1β, and Δ%uric acid of the two groups were compared by means of ANOVAs with SESSION (2 levels, SESSION 1 and SESSION 24), as within-subjects factor, and GROUP (2 levels, CrossFit^®^ and RT), as between-subjects factor. Significant interaction was evaluated through Bonferroni post hoc tests.

The third analysis searched for possible relationships among cortisol, IL-1β, and uric acid log values in the two sessions and groups. To this aim, Pearson’s correlations were applied as follows. POST 1 cortisol values (i.e., dependent variable) were correlated with (1) POST_24 cortisol values—to investigate for possible relationships between values after the first and the last training sessions; (2) POST_1 IL-1β and POST 1 uric acid values—to investigate for possible relationships among salivary measures after training within the same session. The same analysis was repeated considering POST 24 cortisol, POST_1 IL-1β, POST_24 IL-1β, POST_1 uric acid, and POST_24 uric acid, as dependent variables. Moreover, Pearson’s correlations were also applied to evaluate potential relationships among Δ%Cortisol, Δ%IL-1β, and Δ%uric Acid, in SESSION_1 and SESSION_24, in each group. The level of significance was lowered according to Bonferroni’s correction for multiple comparisons (*p* = 0.05/3 = 0.025).

At last, the functional measures were correlated with the salivary measures. Two kinds of analyses were run. In the first analysis, Pearson’s correlation were applied to investigate possible relationship in the two groups among cortisol, IL-1β and uric acid log values at POST 1 and POST 24 (i.e., dependent variables) with baseline measures of 1RM power clean, squat clean, and military press. Furthermore, the percentage changes of functional measures from baseline to ending epochs were computed as follows (endline–baseline)/baseline*100 in each group. The obtained parameters were Δ%power clean, Δ%squat clean, and Δ%military press. In order to evaluate the possible relationship between changes in functional parameters (namely, Δ%power clean, Δ%squat clean, and Δ%military press) and changes in Δ%cortisol, Δ%IL-1β, and Δ%uric Acid, in SESSION 1 and SESSION 24, Pearson’s correlations were applied separately for each group. The level of significance was lowered according to Bonferroni’s correction for multiple comparisons (*p* = 0.05/3 = 0.0167).

The statistical analyses were performed with SPSS (Statistical Package for the Social Sciences, SPSS 20, Chicago, IL, USA). Data are presented as means ± standard error. The probability level taken to indicate significance was *p* < 0.05 (except for multiple comparisons as previously explained). The effect size measures were presented through partial eta squared (η² value), with cut-off points of 0.10, 0.25, 0.40 representing small, medium, and high effect, respectively [35].

## 3. Results

### 3.1. Body Composition and Functional Measures

Table 2 reports the results of the comparisons between participants’ body composition parameters of the two groups, and the results of the maximum repetition tests obtained before (baseline) and after (endline) the 8 weeks of trainings.

Participants’ body weight was comparable in the two groups and did not change after training, as well as the BMI and the lean mass, as shown by the statistical analyses. Fat mass significantly decreased in both groups after the training period, as shown by the significant main effect of EPOCH.

The results of ANOVAs on functional measures (power clean, squat clean, and military presses) showed a significant main effect of EPOCH, indicating a higher performance in both groups after the training period. No differences between group and no significant EPOCH*GROUP interactions were found in any of these parameters.

### 3.2. Cortisol

**Figure 2:**
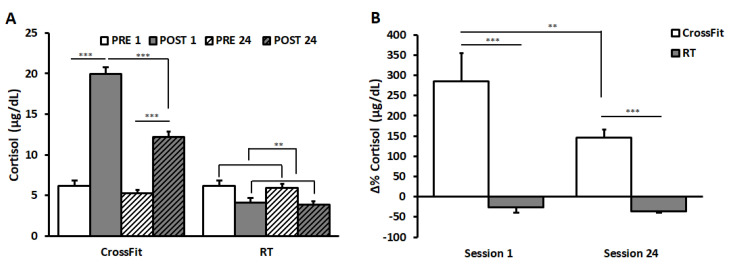
Cortisol value and Δ%cortisol. Cortisol values (panel (**A**)) of CrossFit^®^ group (left) and RT group (right), before (PRE) and after (POST) the first training session (PRE 1, POST 1) and the last training session (PRE 24, POST 24). Δ%Cortisol (panel (**B**)) of CrossFit^®^ group (left) and RT group (right) after the first training session (SESSION 1) and the last training session (SESSION 24). Data are presented as means ± standard error. ** *p* < 0.01; ***p < 0.0001.

Salivary cortisol values of CrossFit^®^ and RT groups are shown in Figure 2A.

The result of ANOVA on data of CrossFit^®^ group showed significant main effects of the factors SESSION (F(1,9) = 96.88, *p* < 0.001, η^2^ = 0.92) and TIME (F(1,9) = 164.74, *p* < 0.001, η^2^ = 0.95), and a significant interaction between them (F(1,9) = 45.66, *p* < 0.001, η^2^ = 0.84). Bonferroni post hoc revealed a significant increase in cortisol values from PRE to POST in both SESSIONS (PRE_1: 6.14 ± 0.67 μg/dL and POST_1: 19.94 ± 0.87 μg/dL; PRE_24: 5.23 ± 0.42 μg/dL and POST_24: 12.19 ± 0.62 μg/dL; *p* = 0.0001). No significant differences appeared between PRE_1 and PRE_24, whilst values in POST_24 were significantly lower than those in POST_1 (*p* = 0.0002).

ANOVA on the RT group showed a significant main effect of TIME (F(1,9) = 25.31, *p* < 0.01, η^2^ = 0.74), indicating a significant decrease from PRE to POST. No differences between sessions and no significant interactions between TIME and SESSION appeared.

Comparing Δ%cortisol values between the two groups (Figure 2B), ANOVA showed a significant main effect of TIME (F(1,18) = 6.83, *p* < 0.05, η^2^ = 0.28) and a significant interaction between TIME and GROUP (F(1,18) = 5.17, *p* < 0.01, η^2^ = 0.22). Bonferroni post hoc revealed significant differences between groups in both sessions (SESSION 1: CrossFit^®^ group 284.78 ± 70.22%, RT group −26.12 ± 13.62%, *p* < 0.0001; SESSION 24 CrossFit^®^ group 145.34 ± 20.47%; RT group −35.82 ± 4.25%, *p* < 0.0001). Furthermore, results showed a significant decrease in the percentage changes in CrossFit^®^ group from SESSION 1 to SESSION 24 (*p* = 0.003).

### 3.3. IL-1β

**Figure 3:**
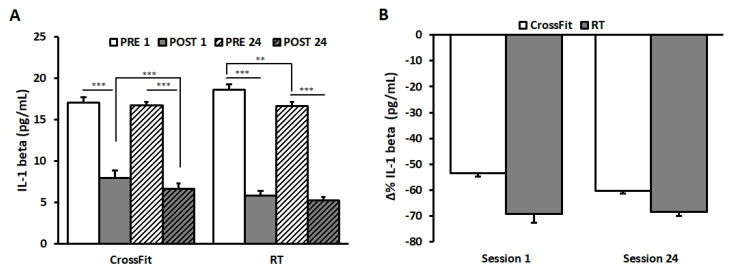
IL-1β values and ∆%IL-1β. IL-1β values (panel (**A**)) of CrossFit^®^ group (left) and RT group (right), measured before (PRE) and after (POST) the first training session (PRE 1, POST 1) and the last training session (PRE 24, POST 24). ∆%IL-1β (panel (**B**)) of CrossFit^®^ group (left) and RT group (right) after the first training session (SESSION 1) and the last training session (SESSION 24). Data are presented as means ± standard error. ** *p* < 0.01; *** *p* < 0.001.

Salivary IL-1β are represented in Figure 3A.

ANOVA on IL-1β values of CrossFit^®^ group showed a significant main effects of the factors SESSION (F(1,9) = 18.17, *p* < 0.01, η^2^ = 0.67) and TIME (F(1,9) = 3001.60, *p* < 0.0001, η^2^ = 0.99), and a significant interaction between them (F(1,9) = 7.11, *p* < 0.05, η^2^ = 0.44). Post hoc tests revealed a significant decrease from PRE to POST in both SESSIONS (PRE_1: 17.04 ± 0.24 pg/mL and POST_1: 7.94 ± 0.27 pg/mL; PRE_24: 16.71 ± 0.24 pg/mL and POST_24: 6.63 ± 0.18 pg/mL; *p* < 0.0001). No significant differences appeared between PRE_1 and PRE_24, whilst values in POST_24 were significantly lower than those in POST_1 (*p* < 0.0001).

ANOVA on IL-1β values of RT group showed significant main effects of the factors SESSION (F(1,9) = 6.87, *p* < 0.05, η^2^ = 0.43) and TIME (F(1,9) = 1206.89, *p* < 0.0001, η^2^ = 0.99) and a significant interaction between them (F(1,9) = 6.74, *p* < 0.05, η^2^ = 0.43). Post hoc tests revealed a significant decrease from PRE to POST in both SESSIONS (PRE_1: 18.60 ± 0.35 pg/mL and POST_1: 5.81 ± 0.69 pg/mL; PRE_24: 16.60 ± 0.37 pg/mL and POST_24: 5.21 ± 0.20 pg/mL; *p* < 0.0001). A significant decrease was observed between PRE_1 and PRE_24 (*p* = 0.001).

The results of the statistical analysis on Δ%IL-1β values between the two groups failed to find significant differences (Figure 3B).

### 3.4. Uric Acid

**Figure 4:**
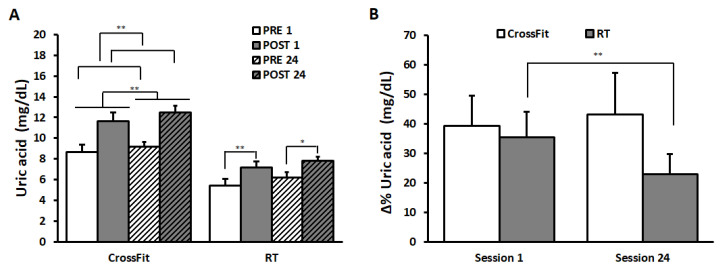
Uric acid values and ∆%uric acid. Uric acid values (panel (**A**)) of CrossFit^®^ group (left) and RT group (right) before (PRE) and after (POST) the first training session (PRE 1, POST 1) and the last training session (PRE 24, POST 24). **∆%**Uric acid (panel (**B**)) of CrossFit^®^ group (left) and RT group (right) after the first training session (SESSION 1) and the last training session (SESSION 24). Data are presented as means ± standard error. * *p* < 0.05; ** *p* < 0.01.

Salivary uric acid values are shown in Figure 4A.

The results of ANOVA on CrossFit^®^ group’s data (PRE_1: 8.68 ± 0.55 mg/dL and POST_1: 11.62 ± 0.36 mg/dL; PRE_24: 9.18 ± 0.53 mg/dL and POST_24: 12.51 ± 0.31 mg/dL) showed a significant main effect of the factor SESSION (F(1,9) = 9.81, *p* < 0.05, η^2^ = 0.52), indicating a significant increase from SESSION 1 to SESSION 24. Further, a significant increase in uric acid values appeared from PRE to POST (TIME: F(1,9) = 20.93, *p* < 0.01, η^2^ = 0.70).

ANOVA on uric acid values of RT group showed a significant main effect of TIME (F(1,9) = 13.58, *p* < 0.01, η^2^ = 0.60) and a significant SESSION*TIME interaction (F(1,9) = 5.08, *p* < 0.05, η^2^ = 0.36). Bonferroni post hoc comparison revealed a significant increase from PRE to POST in both sessions (PRE_1: 5.42 ± 0.41 mg/dL and POST_1: 7.18 ± 0.51 mg/dL; *p* = 0.002; PRE_24: 6.22 ± 0.38 mg/dL and POST_24: 7.79 ± 0.70 mg/dL; *p* = 0.02).

ANOVA comparing Δ%uric acid values between the two groups (Figure 4B) showed a significant main effect of TIME (F(1,18) = 4.54, *p* < 0.05, η^2^ = 0.20) and a significant interaction between TIME and GROUP (F(1,18) = 5.91, *p* < 0.05, η^2^ = 0.25). Bonferroni post hoc revealed a significant decrease in the percentage changes in RT group from SESSION 1 (35.52 ± 8.50%) to SESSION 24 (22.93 ± 6.75%) (*p* = 0.005). No differences appeared in Δ%uric acid values in CrossFit^®^ group (SESSION 1 39.22 ± 10.40%, SESSION 24 43.22 ± 14.06%).

### 3.5. Correlations among Changes in Functional Measures and Cortisol, IL-1β, and Uric Acid

In the RT group only, cortisol levels at POST_24 inversely correlated with all baseline functional measures (power clean: r = −0.85, t = −4.57, *p* = 0.001; squat clean: r = −0.76, t = −3.36, *p* = 0.009; military press: r = −0.82, t = −4.05, *p* = 0.03). IL-1β values at POST_1 positively correlated with baseline military press values (r = 0.66, t = 2–50, *p* = 0.03). Uric acid_log values at POST_24 negatively correlated with baseline squat values (r = −0.74, t = −3.55, *p* = 0.01).

No significant correlations were found between changes in functional measures (Δ%power clean, Δ%squat clean, and Δ%military press) and changes in Δ%cortisol, Δ%IL-1β, and Δ%uric acid in all groups.

### 3.6. Relationship among Cortisol, IL-1β, and Uric Acid Values after Training in the Two Sesions

In the RT group, a significant negative relationship appeared between IL-1β and uric acid values in SESSION 1; namely, the higher the POST 1 IL-1β values, the lower the POST_1 uric acid values (r = −0.80, t = −3.79, *p* = 0.005). Furthermore, POST_1 uric acid values positively correlated with POST_24 uric acid values (r = 0.90, t = 5.91, *p* = 0.0004).

In the CrossFit^®^ group, significant positive relationships were found between Δ%cortisol in SESSION 1 and SESSION 24 (r = 0.80, t = −3.78, *p* = 0.005) and Δ%Uric Acid in SESSION 1 and SESSION 24 (r = 0.95, t = 8.30, *p* = 0.00003).

## 4. Discussion

While CrossFit^®^ and RT trainings induced homogeneous responses in IL-1β levels, heterogeneous responses in cortisol and uric acid levels were documented. Both programs supported a fat mass lowering effect and an anti-inflammatory pattern. In the RT group, an inverse correlation between baseline functional measures and ending cortisol/uric acid levels suggested a favorable downregulation. Instead, the stressful reactions might have been more profound in the CrossFit^®^ group, possibly because of its higher metabolic demand. An overall summary of the major results is offered in Table 3.

The majority of the precedent studies focused on orthopedic (injuries) or psychological aspects of CrossFit^®^, whereas only a few studies analyzed these extreme conditioning programs from a metabolic or hormonal standpoint [14].

The optimal balance between training stimulus and recovery time has always attracted both coaches and athletes, since beneficial adaptations can be translated into enhanced athletic performance. In particular, the purpose of this study was to investigate whether the CrossFit^®^ program and the resistance program affected cortisol, IL-1β, and uric acid levels differently.

- Acute responses were considered as follows:
  - SESSION 1: from PRE to POST
  - SESSION 24: from PRE to POST
- Index of chronic adaptations is expressed as Δ% ((POST-PRE)/PRE*100).

As for cortisol levels, the present results suggest a lesser stress for the RT group with respect to the CrossFit^®^ group.

The primary function of cortisol secretion in response to exercise is to increase the availability of substrates for metabolism [36]. It was shown that cortisol mediates critical physiological processes, which aid exercise capacity and recovery such as promoting proteins in the skeletal muscle proteolysis and lipolysis in adipose tissue [19]. Furthermore, cortisol has been shown to be capable to decrease the production of pro-inflammatory cytokines [37]. Transient cortisol elevations are likely to occur during and immediately following intense exercise [7,13]. CrossFit^®^, utilizing short-intense aerobic intervals, increased significantly from PRE to POST in both SESSIONS, therefore augmenting the availability of substrates for metabolism [11]. In regard to chronic responses, a significant decrease in the percentage changes from SESSION 1 to SESSION 24 (Δ%cortisol) were detected. Cortisol downregulation may represent an enhanced adaptive chronical reaction to exercise. Mangine et al. stated that recreationally active individuals experience systemic responses to protect the muscles and other tissues sensible to glucocorticoids and prevent side effects due to supraphysiological levels of cortisol, which are detrimental for skeletal muscles and, consequently, exercise performance [11]. Present study data are consistent with Poderoso and colleagues, showing an adaptive conditioning effect to high intensity activities [23]. Acute responses of cortisol during resistance exercise span from increases [38] to no changes reported [39]. The obtained findings are in line with previous research, underlying greater acute cortisol responses following metabolically demanding protocols, such as CrossFit^®^ [39] or RT [40]. On the contrary, in the RT group, cortisol levels exhibited a significant decrease from PRE to POST sessions, while no differences were found as per Δ%cortisol between the two sessions. Consistently, earlier reports did not show a significant cortisol response after different strength protocols [41]. Typically, RT is associated with transitory elevated cortisol concentrations, especially when programs are characterized by high intensity volumes and short rest intervals [18]. Therefore, to a relative extent, these current outcomes could be positively interpreted, as they are associated with an immediate decrease. However, in the long-term, as expressed by Δ%, an actual downregulation was not registered.

Skeletal muscle can be considered the largest organ in the human body. Its endocrine-like function is remarkable, as skeletal muscles are able to release several cytokines [42]. In fact, it has been suggested that one of the possible mechanisms linked to the anti-inflammatory effect of physical exercise is the release of IL-6 after an exercise session. On the other hand, IL-1β is the best known immunomodulator factor in response to exercise, and a reduction in the production of IL-1β has been associated with an increase in the release of IL-6, improving the energy supply of skeletal muscles [24]. These observations led to the hypothesis that physical exercise increases anti-inflammatory effects and that it can negatively modulate the immediate inflammatory response at the cellular level. Such an anti-inflammatory response within the bloodstream may induce positive metabolic changes through increased fat oxidation and glucose absorption [43]. Unfortunately, our three-biomarker approach did not include the assessment of IL-6 and certainly it will be considered in the future in order to characterize a more complete picture of the inflammatory network. In the present study, eight weeks of regular power training elicited generally anti-inflammatory effects, which could be protective against chronic systemic low-grade inflammation. In the RT group, basal IL-1β levels were reduced in SESSION 24 with respect to SESSION 1, implying a possibly lower metabolic demand of RT compared to CrossFit^®^ training. However, no significant differences were found in IL-1β levels between the two groups in both sessions. The lack of homogenous responses to these loadings might be related to an extrinsic exercise-depending conditioning mechanism, giving the complexity of multiple abilities required by CrossFit^®^. In this regard, previous studies reported inconclusive findings on the clinical and biochemical traits elicited by strength/power training or HIFT, possibly because of the various methods of assessment, different athletes (sex, age), or simply, different regimes of training [14]. Findings from the present study should be therefore cautiously extrapolated.

Uric acid is an abundant aqueous antioxidant that accounts for about two-thirds of all free radical cleaning activity in human serum [4]. The rise in uric acid is considered a beneficial response to exercise and may result from both increased uric acid production and the lactate-induced inhibition of renal uric acid clearance [44]. Despite the limitation that in this study blood lactate concentration was not measured, we can speculate that the increase in uric acid levels from PRE to POST in both SESSIONS for both groups could have been due to the high calories being used by the body for energy leading to the build-up of lactic acid. Indeed, lactic acid competes with uric acid for excretion during intense training, energy demand, and dehydration, and its antioxidant properties may protect skeletal muscles during high intense exercise [44]. Concerning the amount of changes between PRE and POST values in the two SESSIONS (Δ%uric), the antioxidant role of uric acid may be present after 8 weeks of training in CrossFit^®^ group. Conversely, in the RT group, being a uniform regimen in terms of training load and volume, it is possible that the total antioxidative capacity (pro-oxidant balance [30]) was not maintained. Inconsistent outputs may be indicative of inter-individual response variability, inappropriate selection of workout difficulty, or both. Besides, such a design entailed a hardly comparable workload between the training regimens.

## 5. Conclusions

In this study an ecological, three-biomarker approach related to functional measures was implemented to monitor stress responses in athletes performing strength/power trainings—CrossFit^®^ and RT. Both investigated programs exerted a fat-mass loss and an overall anti-inflammatory effect, which was more pronounced in the RT group, as demonstrated by a chronic downregulation of stress reactions. In fact, CrossFit^®^ appeared to have a more profound catabolic impact than RT, especially as concerns the acute responses. It is likely that the higher intensity with a lesser recovery time between exercises (i.e., a greater metabolic demand) could be the pivotal trigger. Whether this might be physiologically deleterious remains to be ascertained. Certainly, the studied approach might help preventing overtraining circumstances by monitoring biomarkers of stress response. Ultimately, in order to define a complete picture of the dynamics of training and rule out speculations, multiple supplementary assessments should be performed as regards blood lactate and fatigue responses.

A greater understanding of adaptive conditions will depend on future, well-controlled studies, focusing on a multitude of potential stressors, even outside of training, including environmental, social aspects, or cognitive demands.

As such, these preliminary indications might inspire more research alongside additional markers of catabolic status of the participants. Further studies on HIFT or differently stimulated strength/power performances should be warranted, considering the increasing numbers of fitness enthusiasts practicing CrossFit^®^ or other RT regimens. These training programs may be associated to peculiar adaptations that should be favorably harnessed, on a tailored basis, in order to maximize individual metabolic gains.

## Figures and Tables

**Table 1:** Workload components of the training regimes (RT and CrossFit^®^) throughout the 8-week study. (a) Resistance Training; (b) CrossFit^®^.

(a)						
	Week 1–2 (50% 1RM)	Week 3–4 (55% 1RM)	Week 5 (60% 1RM)	Week 6 (65% 1RM)	Week 7 (70% 1RM)	Week 8 (75% 1RM)
**Monday**	3 sets of ▪ 15 reps crunches ▪ 15 reps bicep curls ▪ 15 reps lat. pulldowns	3 sets of ▪ 15 reps crunches ▪ 12 reps bicep curls ▪ 12 reps lat. pulldowns	3 sets of ▪ 15 reps crunches ▪ 10 reps bicep curls ▪ 10 reps lat. pulldowns	3 sets of ▪ 15 reps crunches ▪ 10 reps bicep curls ▪ 10 reps lat. pulldowns	3 sets of ▪ 15 reps crunches ▪ 8 reps bicep curls ▪ 8 reps lat. pulldowns	3 sets of ▪ 15 reps crunches ▪ 8 reps bicep curls ▪ 8 reps lat. pulldowns
**Wednesday**	3 sets of ▪ 15 reps crunches ▪ 15 reps tricep pulldowns ▪ 15 reps bench presses	3 sets of ▪ 15 reps crunches ▪ 12 reps tricep pulldowns ▪ 12 reps bench presses	3 sets of ▪ 15 reps crunches ▪ 10 reps tricep pulldowns ▪ 10 reps bench presses	3 sets of ▪ 15 reps crunches ▪ 10 reps tricep pulldowns ▪ 10 reps bench presses	3 sets of ▪ 15 reps crunches ▪ 8 reps tricep pulldowns ▪ 8 reps bench presses	3 sets of ▪ 15 reps crunches ▪ 8 reps tricep pulldowns ▪ 8 reps bench presses
**Friday**	3 sets of ▪ 15 reps military presses ▪ 15 reps leg extensions ▪ 15 reps reverse leg curls, ▪ 15 reps seated leg presses	3 sets of ▪ 15 reps crunches ▪ 12 reps military presses ▪ 12 reps leg extensions ▪ 12 reps reverse leg curls ▪ 12 reps seated leg presses	3 sets of ▪ 15 reps crunches ▪ 10 reps military presses ▪ 10 reps leg extensions ▪ 10 reps reverse leg curls ▪ 10 reps seated leg presses	3 sets of ▪ 15 reps crunches ▪ 10 reps military presses ▪ 10 reps leg extensions ▪ 10 reps reverse leg curl ▪ 10 reps seated leg presses	3 sets of ▪ 15 reps crunches ▪ 8 reps military presses ▪ 8 reps leg extensions ▪ 8 reps reverse leg curls ▪ 8 reps seated leg presses	3 sets of ▪ 15 reps crunches ▪ 8 reps military presses ▪ 8 reps leg extensions ▪ 8 reps reverse leg curls ▪ 8 reps seated leg presses
**(b)**						
	**1–4 Week**			**5–8 Week**		
**M of AMRAP**	▪ 4′ running ▪ 2′ rest ▪ 4′ jumping rope			▪ 4′ running ▪ 2′ rest ▪ 4′ jumping rope		
**G of AMRAP**	▪ 4′ pull-ups and squats ▪ 2′ rest ▪ 4′ pull-ups and squats			▪ 4′ pull-ups and squats ▪ 2′ rest ▪ 4′ pull-ups and squats		
**W of AMRAP**	▪ 4′ front squats and kettlebell swings at 50–60% 1RM ▪ 2′ rest ▪ 4′ front squats and kettlebell swings at 50–60% 1RM			▪ 4′ front squats and kettlebell swings at 65–75% 1RM ▪ 2′ rest ▪ 4′ front squats and kettlebell swings at 65–75% 1RM		

M = metabolic conditioning exercises; G = gymnastics exercises; W = weightlifting exercises; AMRAP = “as many repetitions as possible” routine.

**Table 2:** Participants’ anthropometric characteristics and results of testing procedures before and after the training regimes (mean ± SD).

Variables	CrossFit^®^ Group		RT Group		Statistics
	BASELINE	ENDLINE	BASELINE	ENDLINE	
Weight (kg)	72.59 ± 5.85	71.31± 6.45	75.38 ± 8.32	75.42 ± 7.87	N.S.
BMI (kg/m^2^)	23.30 ± 0.97	22.87± 0.98	24.46 ± 1.91	24.39 ± 1.89	N.S.
Lean Mass (kg)	63.02 ± 5.84	62.75± 5.69	64.30± 6.71	64.85± 6.28	N.S.
Fat Mass (%)	8.68 ± 4.20	7.37 ± 3.12	10.12 ± 2.91	9.28 ± 2.87	EPOCH: F(1,18) = 9.08, *p* = 0.007, η^2^ = 0.34
1RM Power Clean (kg)	74.80 ± 9.81	80.20 ± 8.30	81.3 ± 8.30	85.20 ± 8.18	EPOCH: F(1,18) = 15.65, *p* = 0.001, η^2^ = 0.47
1RM Squat Clean (kg)	75.60 ± 6.35	87.40 ± 15.23	90.30± 10.34	96.00 ± 19.26	EPOCH: F(1,18) = 17.74, *p* = 0.001, η^2^ = 0.50
1RM Military Press (kg)	45.10 ± 8.57	51.60 ± 8.37	53.10 ± 9.89	57.10 ± 11.63	EPOCH: F(1,18) = 23.49, *p* < 0.0001, η^2^ = 0.57

N.S. = not significant.

**Table 3:** Synopsis of the major results elicited by the two conditioning training programs.

	ACUTE (POST–PRE)		CHRONIC (Δ%)	
	CrossFit^®^	RT	CrossFit^®^	RT
Cortisol	↑	↓	↓	=
IL-1β	↓	↓	=	=
Uric Acid	↑	↑	=	↓
